# Longitudinal motor function and biomarker correlates in treated adult spinal muscular atrophy: a single-center cohort study

**DOI:** 10.3389/fneur.2026.1870339

**Published:** 2026-06-11

**Authors:** Chikashi Yano, Masahiro Ando, Akiko Yoshimura, Yujiro Higuchi, Yoshikatsu Noda, Jun-Hui Yuan, Tomonori Nakamura, Takahiro Hobara, Risa Nagatomo, Fumikazu Kojima, Mika Yuji, Yu Hiramatsu, Satoshi Nozuma, Akihiro Hashiguchi, Yusuke Sakiyama, Hiroshi Takashima

**Affiliations:** 1 Department of Neurology and Geriatrics, Kagoshima University Graduate School of Medical and Dental Sciences, Kagoshima, Japan; 2 Division of Neurology, Kobe University Graduate School of Medicine, Kobe, Japan; 3 Department of Neurology, National Hospital Organization Okinawa Hospital, Okinawa, Japan

**Keywords:** biomarkers, hybrid SMN alleles, nusinersen, risdiplam, *SMN2* copy number, spinal muscular atrophy

## Abstract

**Background:**

Long-term clinical trajectories and accessible biomarkers in adult spinal muscular atrophy (SMA) remain insufficiently characterized under real-world treatment conditions. We conducted a single-center longitudinal study to evaluate motor function, genotype–phenotype relationships, and clinically accessible biomarkers in treated adults with SMA.

**Methods:**

Twenty-three Japanese adults with genetically confirmed 5q-SMA treated with nusinersen or risdiplam (2018–2025) underwent repeated assessments of Revised Upper Limb Module (RULM), Hammersmith Functional Motor Scale Expanded (HFMSE), and laboratory, physiological, and electrophysiological measures. Associations were explored using correlation analyses, linear mixed-effects models, and exploratory machine-learning approaches.

**Results:**

Higher *SMN2* copy number was associated with later onset and milder severity. Hybrid *SMN* alleles showed heterogeneous patterns depending on copy-number context. Longitudinally (mean follow-up 57 months), motor scores often improved or stabilized during the first year after treatment initiation, followed by plateau or gradual decline in some patients. In mixed-effects models, vital capacity (%VC) was independently associated with RULM, while other biomarkers showed consistent associations with motor scores. Exploratory machine-learning suggested that ulnar compound muscle action potential (CMAP) amplitude and creatine kinase (CK) contributed to model predictions, although baseline features did not reliably predict short-term motor changes.

**Conclusion:**

In treated adults with SMA, simple clinical measures such as %VC, CK, ulnar CMAP amplitude are associated with motor status and may support routine monitoring, while prediction of treatment responsiveness remains challenging.

## 1 Introduction

Spinal muscular atrophy (SMA) is an autosomal recessive motor neuron disease most often caused by homozygous deletions or mutations in the survival motor neuron 1 (*SMN1*) gene, which encodes the SMN protein. Although its paralog *SMN2* also produces SMN protein, approximately 90% of its transcripts lack exon 7 due to alternative splicing, resulting in limited production of functional SMN protein. The number of *SMN2* copies is a well-established genetic modifier that strongly influences age at onset and disease severity (1, 2). Disease-modifying therapies such as nusinersen and risdiplam, which increase SMN protein expression, have recently transformed the natural history of SMA (3–6). However, in adult patients, the long-term clinical course under these treatments remains incompletely characterized. In particular, whether early treatment-related improvements are sustained over time is not fully understood. Current standards for evaluating treatment response, including the Revised Upper Limb Module (RULM) and the Hammersmith Functional Motor Scale Expanded (HFMSE), are time-consuming, require trained examiners, and can be physically demanding for patients (7, 8). These limitations highlight the need for simpler and more accessible surrogate markers that reflect motor function in routine clinical practice.

In addition, genotype–phenotype relationships in adults with SMA remain insufficiently defined. Although *SMN2* copy number is a key determinant of disease severity, additional modifiers such as hybrid *SMN* genes–defined by exon 7 derived from *SMN2* and exon 8 from *SMN1*–have been reported, particularly in Japanese populations (9). However, their clinical significance remains uncertain.

To address these gaps, we conducted a longitudinal observational study in adults with SMA treated with nusinersen or risdiplam. Our objectives were threefold: (1) to characterize the long-term clinical trajectories under real-world treatment conditions; (2) to explore genotype–phenotype relationships including *SMN2* copy number and hybrid alleles; and (3) to identify accessible clinical biomarkers associated with motor function, and to evaluate their potential utility using both statistical and machine-learning approaches.

## 2 Materials and methods

### 2.1 Study design and ethics

This was a single-center, longitudinal observational cohort study. Clinical, laboratory, and physiological/electrophysiological parameters were measured at multiple time points per patient and analyzed using correlation analysis, linear mixed-effects models (LMMs), and machine learning-based regression models. Owing to the rarity of 5q-SMA in adults, all consecutive eligible cases during the study period were included. No formal *a priori* sample-size calculation was performed. Analyses were therefore treated as exploratory, with an emphasis on effect sizes and 95% confidence intervals. This study was approved by the institutional ethics board of Kagoshima University and conducted in accordance with the Declaration of Helsinki (World Medical Association, 2013).

### 2.2 Participants and treatment

All consecutive genetically confirmed 5q-linked SMA patients who received nusinersen or risdiplam at Kagoshima University Hospital between January 2018 and March 2025 were included. No exclusion criteria were applied, and all eligible patients were enrolled (*N* = 23). Nusinersen was administered at a dose of 12 mg via intrathecal injection, with loading doses on days 0, 30, and 90, followed by maintenance doses every 6 months (10). Patients who transitioned to risdiplam (5 mg/day orally) were evaluated every 6 months thereafter (11). Some patients subsequently returned to nusinersen treatment. All clinical and laboratory data were collected retrospectively from medical records, with follow-up continuing through March 2025.

### 2.3 Clinical and genotype classification

SMA type was classified according to age at symptom onset, based on established criteria (12). All patients had homozygous deletions of *SMN1* exons 7 and/or 8. *SMN2* copy number and the presence of hybrid *SMN* alleles were determined by multiplex ligation-dependent probe amplification.

### 2.4 Motor and biomarker assessments

Motor function was evaluated using the HFMSE and RULM (7, 8). The 6-min walk test (6MWT) was also performed in ambulant patients. Clinically meaningful improvement was defined as an increase of ≥3 points in HFMSE or ≥2 points in RULM, consistent with prior reports (6, 13, 14). Candidate biomarkers included laboratory markers [creatine kinase (CK), serum creatinine (Cr), and creatine] and physiological/electrophysiological parameters [vital capacity (%VC), ulnar compound muscle action potential (CMAP) amplitude, and decremental response on 3 Hz repetitive nerve stimulation]. %VC was used to assess trunk and respiratory muscle function, while the 6MWT reflected ambulatory endurance. CMAP decrement was included because it has been observed in SMA and is clinically relevant in amyotrophic lateral sclerosis (15–20). Electrophysiological recordings were obtained with the Neuropack X1 system (Nihon Kohden, Tokyo, Japan) using established methods (21). Slow vital capacity (%VC) was measured by spirometry in the seated position with a nose clip and expressed as a percentage of predicted values.

#### 2.4.1 Data completeness and missingness

As a real-world longitudinal study, some follow-up visits lacked motor scale and/or laboratory/electrophysiological data. The main reasons were as follows: (i) the patient’s condition precluded testing, and (ii) deferral of in-person visits during the COVID-19 pandemic. Missingness was considered plausibly missing at random, conditional on patient and time. Analytical handling of missing data is described in section 2.5.

### 2.5 Statistical and machine-learning analysis

Analyses were performed using R 4.3.2 (RStudio) and Python 3.11 (Colab). Repeated measures were analyzed with random-intercept LMMs or subject-wise cross-validation. For concurrent score prediction (RULM/HFMSE), leave-one-subject-out cross-validation (LOSO) was used at the patient level, with all preprocessing/tuning performed within the training fold. Performance was reported as out-of-fold *R*^2^, root mean square error (RMSE, points), and mean absolute error (MAE, points). For short-term change (*Δ* at 9 months), nested cross-validation (outer fivefold; inner grid search) was used, and mean ± SD values were reported across outer folds.

#### 2.5.1 Correlations

Pearson correlations were computed between motor scores (RULM, HFMSE) and candidate parameters (CK, Cr, creatine, %VC, ulnar CMAP amplitude, ulnar nerve decremental response, 6MWT) using pairwise deletion (pairwise-complete observations).

#### 2.5.2 Linear mixed-effects models

To identify independent predictors of motor function, separate random-intercept models were fitted for RULM and HFMSE (patient ID as the random effect). Missing predictor values were imputed within a patient in the order: linear interpolation → forward-fill → backward-fill. Outcomes were not imputed. Fixed effects were pre-specified by correlation screening (|*r*| ≥ 0.4) and limited to %VC, CK, Cr, creatine, and ulnar CMAP amplitude. The 6MWT was excluded *a priori* because it overlaps with the motor outcomes. Models were adjusted for month (centered) and treatment, estimated by restricted maximum likelihood (REML), and reported with coefficients and 95% confidence intervals.

#### 2.5.3 Machine learning

Using the same imputed dataset, Ridge, Elastic Net, and Random Forest models were trained to predict RULM and HFMSE, including all predictors excluding the 6MWT. No correlation filter was applied. Evaluation used LOSO with fold-internal preprocessing/tuning to prevent data leakage (median imputation and standardization within training folds). Performance was summarized by R^2^, RMSE, and MAE. Random Forests were interpreted with SHapley Additive exPlanations (SHAP).

#### 2.5.4 Change-over-time prediction

Nine-month changes in motor function (ΔRULM, ΔHFMSE) were modeled using baseline predictors (%VC, CK, Cr, creatine, ulnar CMAP amplitude, ulnar nerve decremental response, plus the corresponding baseline motor score). The outcome was the motor score recorded at Month 9 after treatment initiation, corresponding to the first post-loading assessment. Each patient contributed one row, and outcomes were not imputed. Models were evaluated with nested cross-validation (outer fivefold; inner grid search), with all preprocessing performed within training folds to avoid leakage. This resulted in 18 evaluable patients for both ΔRULM and ΔHFMSE. Further methodological details are provided in Supplementary methods.

## 3 Results

### 3.1 Characteristics of the study population

#### 3.1.1 Demographics and clinical subtypes

A total of 23 adult patients (15 males and 8 females) with genetically confirmed SMA were enrolled in this study. The median age at treatment initiation was 42 years (range, 17–72), and the median age at symptom onset was 3 years (range, 1–20). At enrolment, the median disease duration was 23 years (range, 14–69). By clinical subtype, six patients had SMA type II and 17 had SMA type III. Six patients were ambulant at baseline, while the remaining 17 were non-ambulant. One patient required a percutaneous endoscopic gastrostomy tube, and three required ventilatory support, including two who used noninvasive ventilation only during the night. Two patients had previously undergone spinal fusion surgery for scoliosis.

#### 3.1.2 Genotype classification

**Figure 1:**
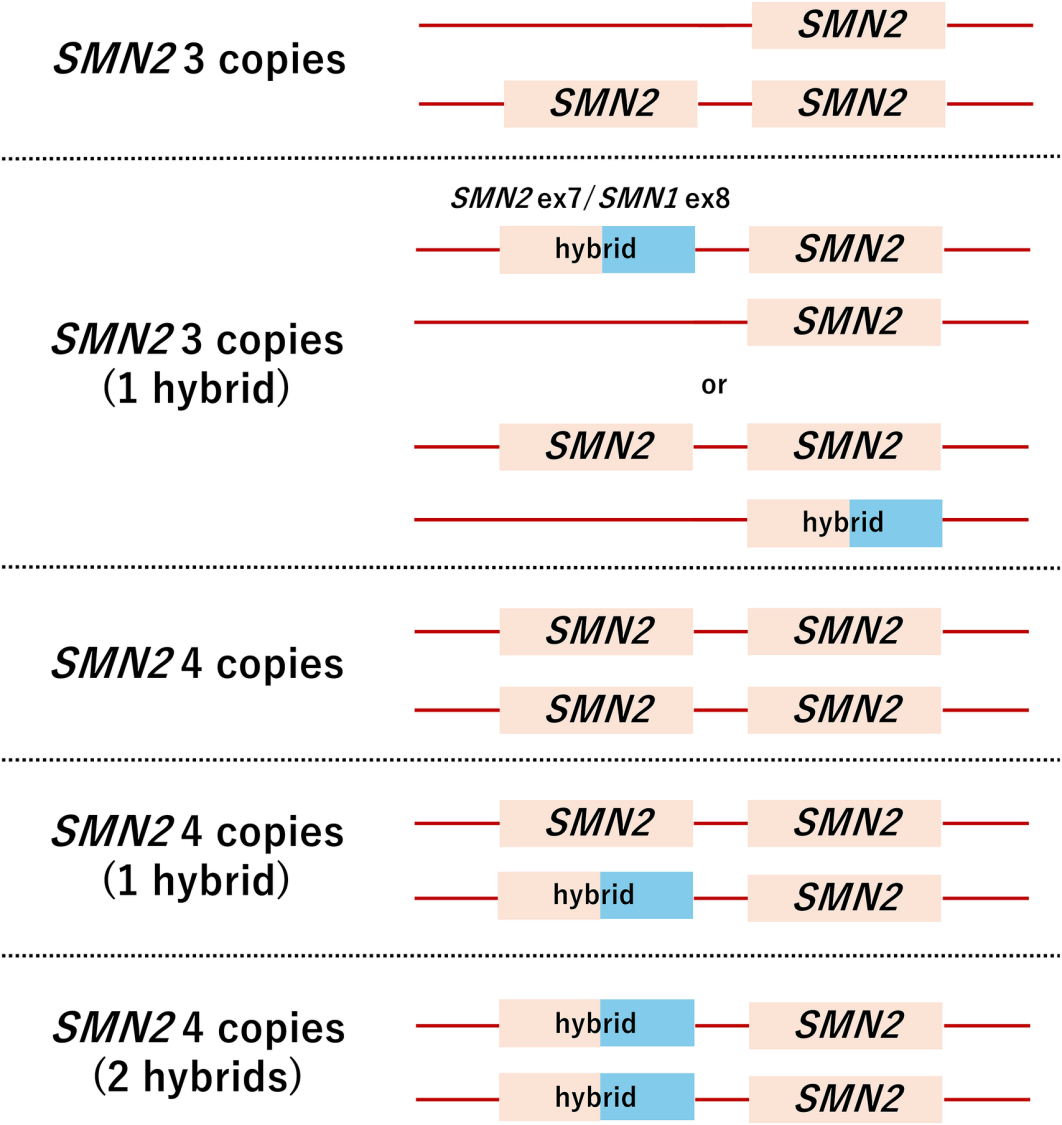
Genomic structures of variant *SMN2* genes. Five *SMN2*-related genomic structures were identified in this cohort, comprising combinations of standard *SMN2* alleles and hybrid alleles. In the schematic diagram, blue boxes represent exon 8 derived from the *SMN1*, while orange boxes represent exons from *SMN2*. Hybrid alleles contain exon 7 from *SMN2* and exon 8 from *SMN1*, and are illustrated accordingly. These structural variations were used to classify genotypes and examine genotype–phenotype correlations.

All patients carried homozygous deletions in exons 7 and/or 8 of the *SMN1* gene. Genotyping based on *SMN2* copy number and hybrid *SMN* alleles revealed five distinct categories. Four patients had three *SMN2* copies without hybrid alleles, and another four had three *SMN2* copies, one of which was a hybrid allele. Eleven patients carried four *SMN2* copies without hybrid alleles, while three had four *SMN2* copies, one of which was a hybrid allele. One patient had four *SMN2* copies, two of which were hybrid alleles. These structural classifications are illustrated in Figure 1 and were used for genotype–phenotype correlation analyses (Figure 1).

#### 3.1.3 Baseline clinical and biomarker profiles

**Figure 2:**
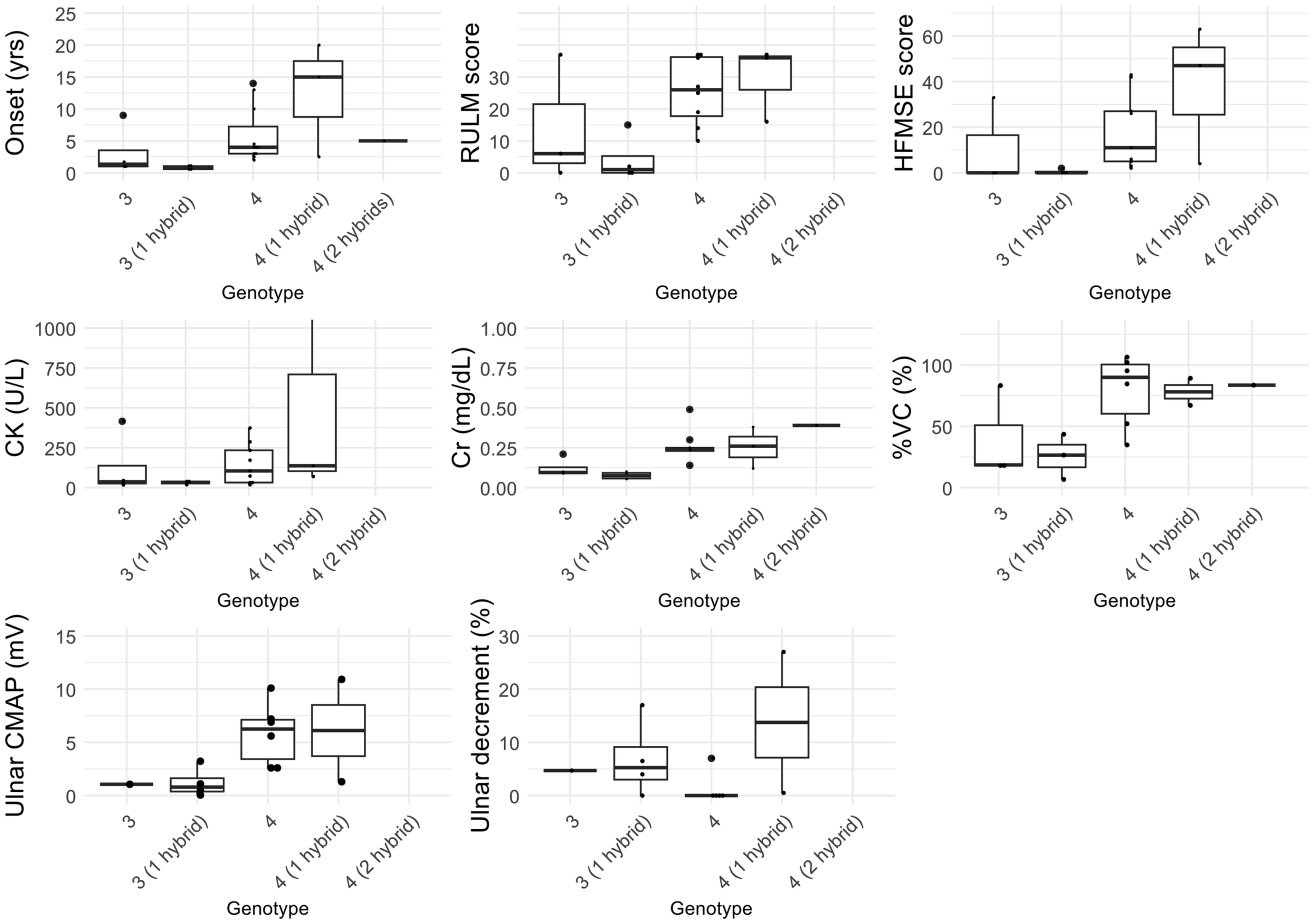
Genotype-specific distributions of clinical, laboratory, and physiological/electrophysiological parameters. Boxplots show the distribution of clinical scores (age at onset, RULM, HFMSE), laboratory markers (CK, Cr, creatine), and physiological/electrophysiological parameters (%VC, ulnar nerve CMAP amplitude, ulnar nerve decremental response), stratified by *SMN2* genotype. Each group represents a distinct *SMN2* copy number and hybrid configuration: three *SMN2* copies without hybrids (*n* = 4); three copies with one hybrid (*n* = 4); four copies without hybrids (*n* = 11); four copies with one hybrid (*n* = 3), and four copies with two hybrids (*n* = 1). RULM, Revised Upper Limb Module; HFMSE, Hammersmith Functional Motor Scale Expanded; CK, creatine kinase; Cr, serum creatinine; %VC, vital capacity; CMAP, compound muscle action potential.

Baseline RULM scores were available for 18 patients [median 17.5; interquartile range (IQR), 7–36] and HFMSE scores for 19 patients (median 5; IQR, 1–30). For genotype-stratified descriptions, the single case with four *SMN2*-related copies, including two hybrid alleles (*n* = 1), was excluded from between-group summaries. Across the remaining categories, baseline clinical severity and age at onset followed a graded pattern from milder/later-onset to more severe/earlier-onset in the following order: four copies with one hybrid, four copies without hybrids, three copies without hybrids, and three copies with one hybrid. Physiological and electrophysiological measures showed concordant variation, with higher ulnar CMAP amplitudes and greater %VC observed in the milder groups (Table 1 and Figure 2).

**Table 1:** Baseline genetic, clinical, and physiological/electrophysiological characteristics by *SMN2*/hybrid genotype.

*SMN2* genotype	3 copies (*N* = 4)	3 copies (1 hybrid) (*N* = 4)	4 copies (*N* = 11)	4 copies (1 hybrid) (*N* = 3)	4 copies (2 hybrids) (*N* = 1)
Sex (M: F) (n)	1:3	4:0	7:4	2:1	1:0
Age (years)	33.5 (26.5–41.5)	29.5 (24.5–33.5)	67 (59.5–68.5)	38 (38–40)	20
Onset (years)	1.35 (1–3.525)	0.8 (0.575–1.05)	4 (3–7.25)	15 (8.75–17.5)	5
RULM	6 (3–21.5) *N* = 3	1 (0–5.25) *N* = 4	26 (17.75–36.25) *N* = 8	36 (26–36.5) *N* = 3	NA
HFMSE	0 (0–16.5) *N* = 3	0 (0–0.5) *N* = 4	11 (5–27) *N* = 9	47 (25.5–55) *N* = 3	NA
CK (U/L)	37 (26–137.75) *N* = 4	33.5 (26.25–38.25) *N* = 4	105 (32–234) N = 9	137 (103.5–709.5) *N* = 3	2,180 N = 1
Cr (mg/dL)	0.1 (0.09–0.13) *N* = 4	0.07 (0.06–0.09) *N* = 4	0.24 (0.23–0.25) *N* = 9	0.26 (0.19–0.32) *N* = 3	0.39 *N* = 1
Creatine (mg/dL)	NA	1.59 (1.55–1.64) *N* = 2	NA	NA	NA
%VC (%)	18.5 (18.05–50.85) *N* = 3	26 (16.6–35.0) *N* = 3	89.85 (60.2–100.3) *N* = 6	78.05 (72.53–83.57) *N* = 2	83.5 *N* = 1
Ulnar CMAP (mV)	1.06 *N* = 1	0.8 (0.38–1.63) *N* = 4	6.25 (3.43–7.12) *N* = 6	6.11 (3.7–8.51) *N* = 2	NA *N* = 0
Ulnar decrement (%)	4.7 *N* = 1	5.25 (3–9.12) *N* = 4	0 (0–0) *N* = 5	13.75 (7.12–20.38) *N* = 2	NA *N* = 0
6MWT (m)	NA *N* = 0	NA *N* = 0	65 *N* = 1	424 (397–451) *N* = 2	NA *N* = 0

Each column represents a patient group categorized by *SMN2* gene copy number and the number of hybrid *SMN2* genes. Data presented as median (interquartile range). M, male; F, female; RULM, Revised Upper Limb Module; HFMSE, Hammersmith Functional Motor Scale Expanded; CK, creatine kinase; Cr, serum creatinine; %VC, vital capacity; CMAP, compound muscle action potential; 6MWT, 6-min walk test; NA, not assessed.

### 3.2 Longitudinal clinical course

#### 3.2.1 Treatment history and follow-up

**Figure 3:**
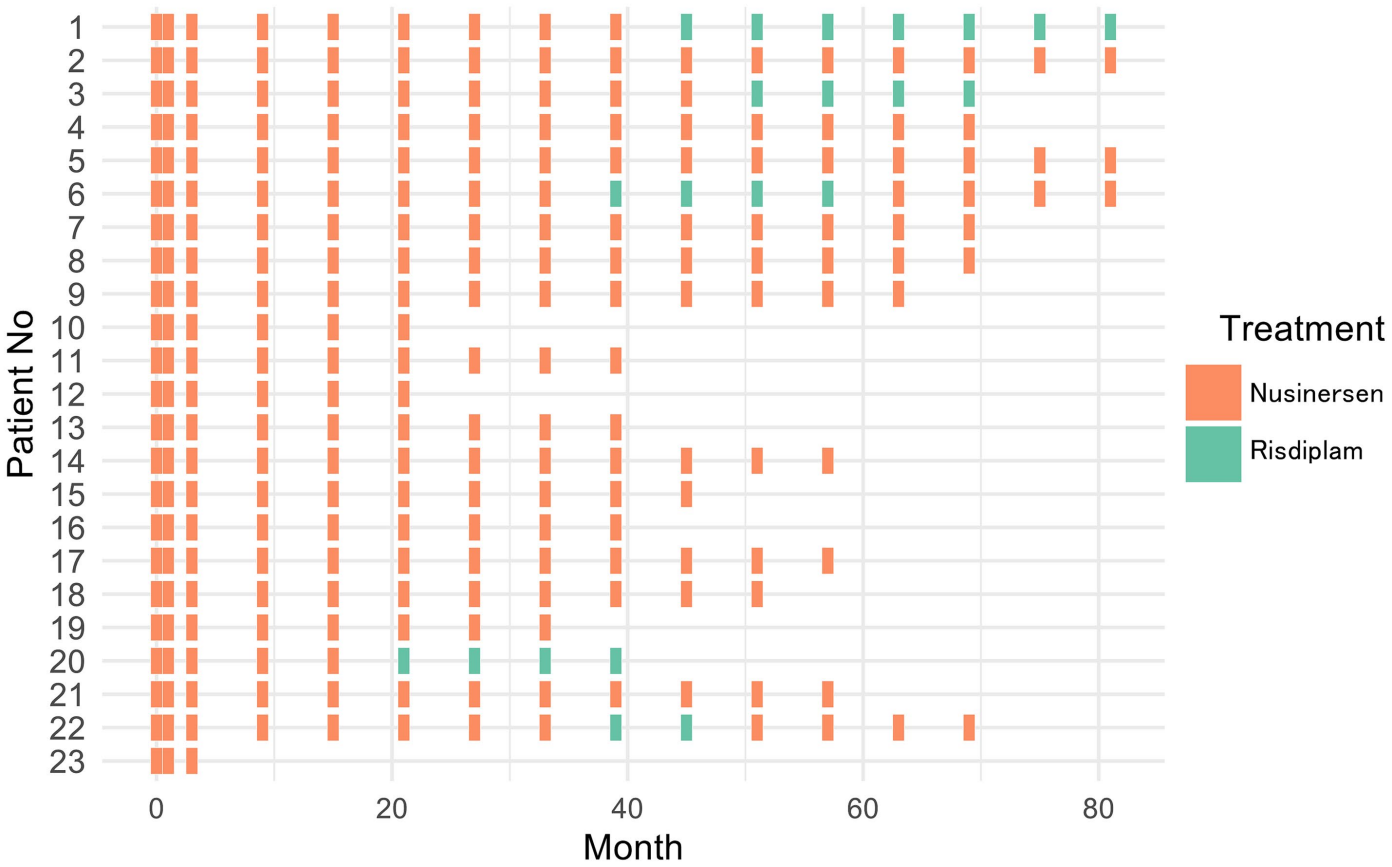
Treatment and follow-up. Nusinersen was administered with loading doses at 0, 1, and 3 months, followed by maintenance dosing every 6 months with clinical evaluations at each injection. For patients who were switched to risdiplam, evaluations were performed every 6 months. Orange bars indicate nusinersen treatment, and green bars indicate risdiplam. Five patients transitioned to risdiplam during the study period, and two of these later reverted to nusinersen. The mean follow-up duration was 57 months, with a maximum of 81 months.

All patients were initiated on nusinersen therapy between January 2018 and January 2024. Clinical and biomarker assessments were performed at baseline, at one and 3 months, and every 6 months thereafter. The mean follow-up period was 57 months, with a maximum of 81 months. During follow-up, five patients were switched from nusinersen to risdiplam, and two of these later returned to nusinersen (Figure 3). Reasons for switching included avoidance of hospital visits during the COVID-19 pandemic, difficulty with intrathecal administration due to spinal deformity, and preference for oral treatment or perceived lack of efficacy. Re-initiation of nusinersen occurred due to adverse effects such as diarrhea or insufficient response to risdiplam. A representative case with sequential treatment is shown in Supplementary file patient 6.

#### 3.2.2 Temporal changes in motor function

**Figure 4:**
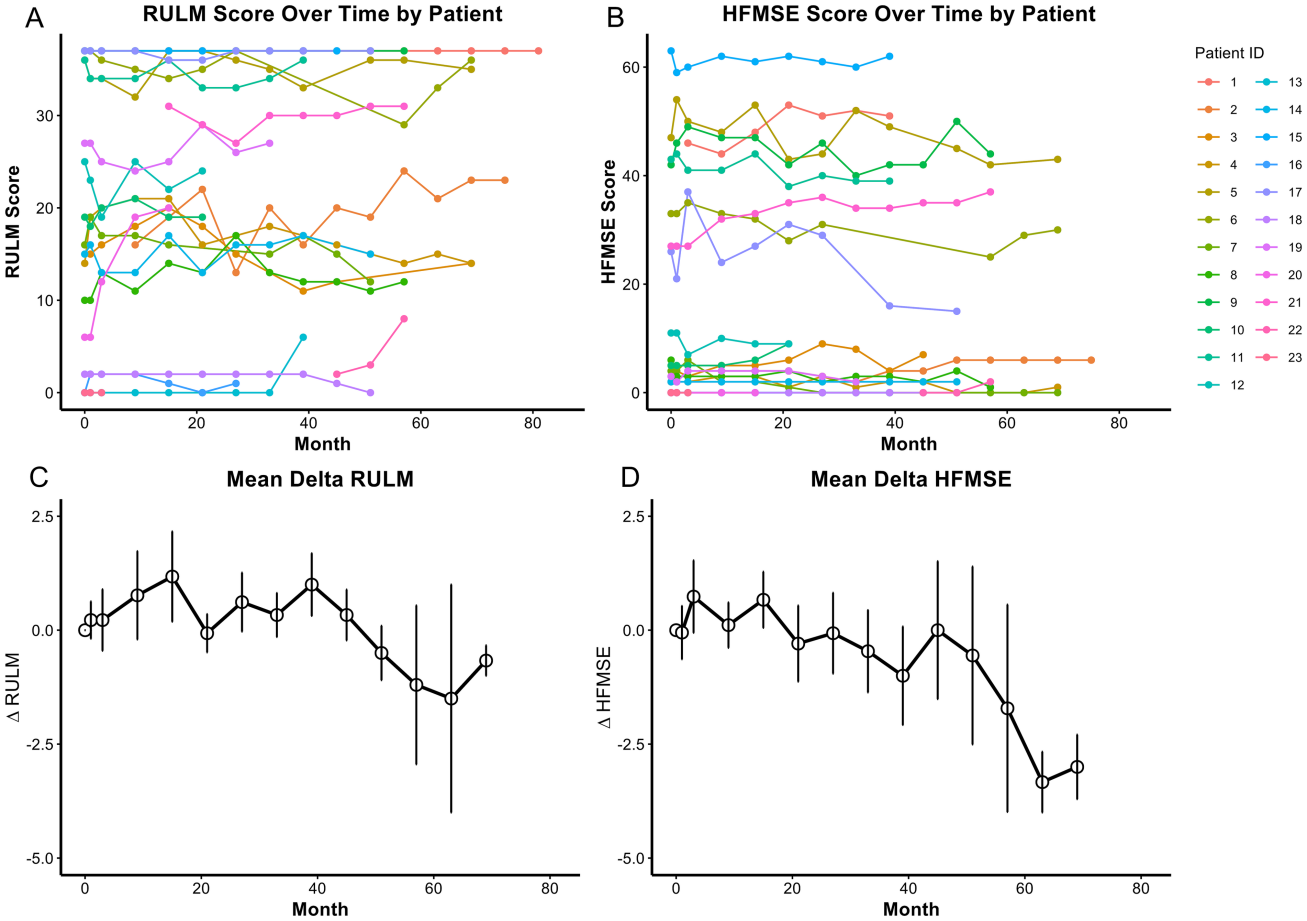
RULM and HFMSE scores after treatment initiation. **(A,B)** Individual trajectories of RULM and HFMSE scores over time. **(C,D)** Mean changes from baseline (ΔRULM and ΔHFMSE) at each follow-up period. RULM, Revised Upper Limb Module; HFMSE, Hammersmith Functional Motor Scale Expanded.

Longitudinal evaluation of HFMSE and RULM scores demonstrated heterogeneous trajectories across patients (Figures 4A,B). Among the 18 patients with both baseline and 9-month follow-up data, six showed clinically meaningful improvement, six remained stable, and six showed declines. Mean changes in motor scores peaked within approximately 20 months after treatment initiation, followed by gradual decline, suggesting a plateau or attenuation of therapeutic effects over time (Figures 4C,D).

### 3.3 Association between biomarkers and motor function

#### 3.3.1 Correlation analysis

Correlations between motor/functional scores and candidate biomarkers are summarized in Table 2. RULM showed strong correlations with Cr (*r* = 0.775), %VC (*r* = 0.829), and ulnar CMAP amplitude (*r* = 0.832), and a moderate correlation with CK (*r* = 0.498). Serum creatine demonstrated a weak negative correlation (*r* = −0.434). Similarly, HFMSE showed positive correlations with CK (*r* = 0.604), Cr (*r* = 0.763), ulnar CMAP amplitude (*r* = 0.783), and %VC (*r* = 0.696), while serum creatine showed a weak negative correlation (*r* = −0.494).

**Table 2:** Correlation matrix of motor function scores and clinical parameters.

Correlation coefficient	RULM	HFMSE	CK	Cr	Creatine	%VC	Ulnar CMAP	Ulnar decrement	6MWT
RULM	1	0.843	**0.498**	**0.775**	**−0.434**	**0.829**	**0.832**	−0.273	0.317
HFMSE	0.843	1	**0.604**	**0.763**	**−0.494**	**0.696**	**0.783**	−0.335	0.839
CK	0.498	0.604	1	0.434	−0.052	0.441	0.418	−0.098	0.15
Cr	0.775	0.763	0.434	1	−0.286	0.727	0.699	−0.339	0.352
Creatine	−0.434	−0.494	−0.052	−0.286	1	−0.17	−0.482	0.411	−0.276
%VC	0.829	0.696	0.441	0.727	−0.17	1	0.697	−0.245	−0.122
Ulnar CMAP	0.832	0.783	0.418	0.699	−0.482	0.697	1	−0.306	0.774
Ulnar decrement	−0.273	−0.335	−0.098	−0.339	0.411	−0.245	−0.306	1	−0.216
6MWT	0.317	0.839	0.15	0.352	−0.276	−0.122	0.774	−0.216	1

Pearson correlation coefficients are shown among motor and functional measures (RULM, HFMSE, and 6MWT), laboratory biomarkers (CK, Cr, and creatine), and physiological/electrophysiological measures (%VC, ulnar CMAP amplitude, and ulnar decrement on repetitive nerve stimulation). RULM, Revised Upper Limb Module; HFMSE, Hammersmith Functional Motor Scale Expanded; CK, creatine kinase; Cr, serum creatinine; %VC, vital capacity; CMAP, compound muscle action potential; 6MWT, 6-min walk test. Bold and underlined values indicate variables with an absolute correlation coefficient (|r|) ≥ 0.4 that were selected for inclusion in the linear mixed-effects models.

#### 3.3.2 Linear mixed-effects modeling

To investigate independent predictors of motor function, random-intercept LMMs were constructed using variables pre-specified by correlation screening (coefficient matrix, |*r*| ≥ 0.4) with RULM or HFMSE as the outcome. Candidate predictors included CK, Cr, creatine, %VC, and ulnar CMAP amplitude, with month (centered) and treatment included as adjustment covariates. In the RULM model, %VC was identified as a statistical significant predictor (*β* = 0.186, *p* < 0.001), while CK, Cr, creatine, and ulnar CMAP amplitude were not significant. In the HFMSE model, no predictors reached statistical significance. However, Cr (*β* = 14.447, *p* = 0.059) and ulnar CMAP amplitude (*β* = 0.411, *p* = 0.069) showed borderline associations. Adjustment covariates (time and treatment) were included but not interpreted. The results are summarized in Table 3.

**Table 3:** Linear mixed-effects models identifying predictors of motor function.

Predictor	Coefficient	Std. error	*z*-value	*p*-value	95% CI
RULM					
Intercept	7.213	3.37	2.14	0.032	0.608–13.818
%VC	0.186	0.039	4.794	<0.001	0.11–0.262
CK	−0.001	0.002	−0.301	0.763	−0.004–0.003
Cr	3.327	6.307	0.527	0.598	−9.034–15.688
Creatine	−0.02	1.162	−0.018	0.986	−2.298–2.258
Ulnar CMAP	0.245	0.192	1.279	0.201	−0.131–0.621
HFMSE					
Intercept	7.323	5.477	1.337	0.181	−3.411–18.057
%VC	0.021	0.051	0.417	0.677	−0.079–0.122
CK	−0.002	0.002	−0.888	0.374	−0.006–0.002
Cr	14.447	7.645	1.89	0.059	−0.536–29.43
Creatine	1.583	1.206	1.313	0.189	−0.78–3.946
Ulnar CMAP	0.411	0.226	1.815	0.069	−0.033–0.855

Random-intercept mixed-effects models (random intercept for patient ID) were estimated using restricted maximum likelihood (REML). Fixed effects were pre-specified by correlation screening (|*r*| ≥ 0.4): vital capacity (%VC), creatine kinase (CK), serum creatinine (Cr), creatine, and ulnar CMAP amplitude. Models were additionally adjusted for month (centered) and treatment. Results are reported as β (SE), Wald z, p-value, and 95% CI. Missing values in longitudinal series were imputed within patient (linear interpolation → forward-fill → back-fill). Final analytic samples were: RULM, 181 observations from 20 patients; HFMSE, 190 observations from 20 patients. Units: %VC, %; CK, U/L; Cr, mg/dL; creatine, mg/dL; ulnar CMAP, mV. RULM, Revised Upper Limb Module; HFMSE, Hammersmith Functional Motor Scale Expanded; Std, standard; CI, confidence interval; %VC, vital capacity; CK, creatine kinase; Cr, serum creatinine; CMAP, compound muscle action potential.

#### 3.3.3 Machine-learning models and SHAP interpretation

**Figure 5:**
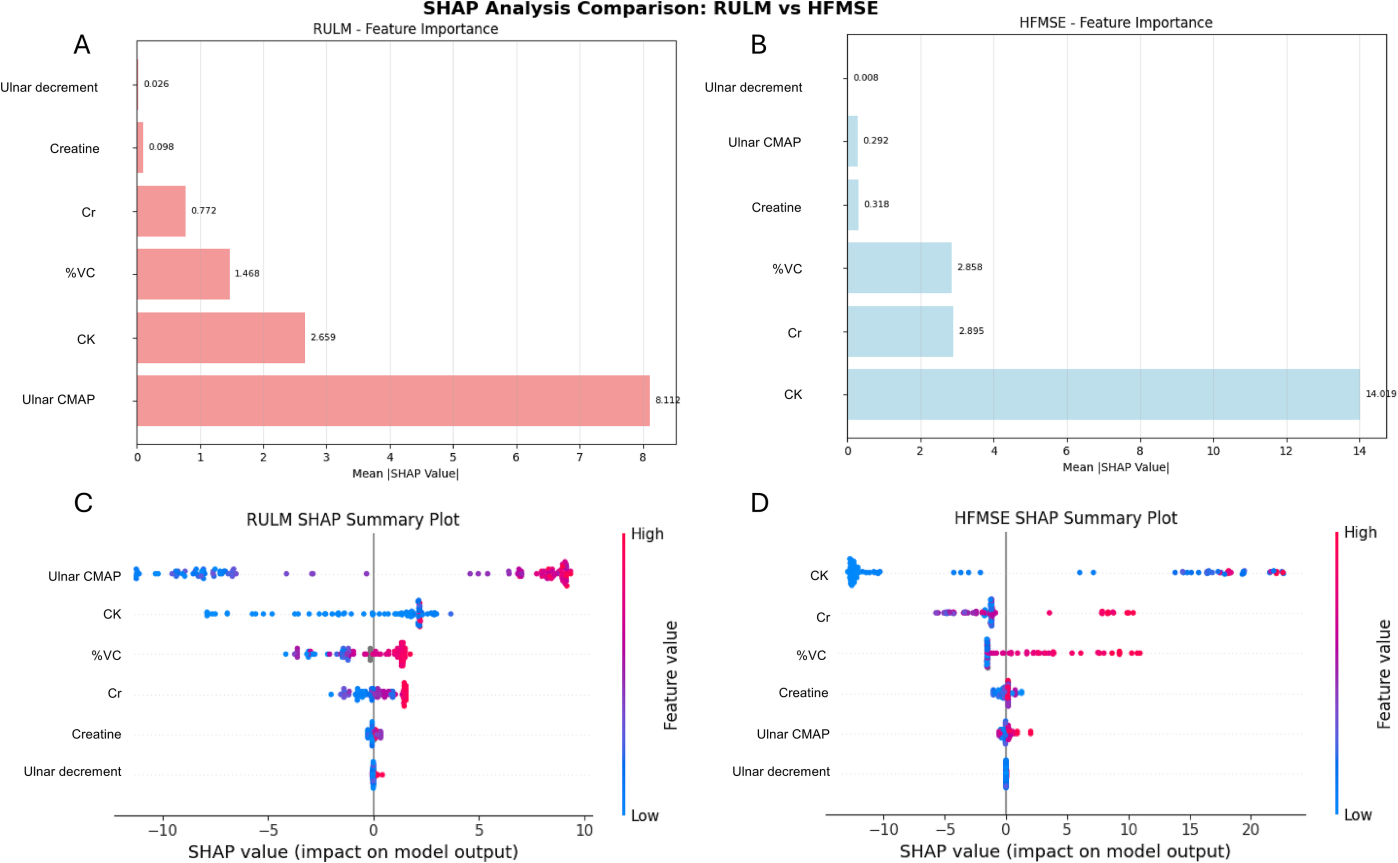
SHAP analyses for random forest models predicting RULM and HFMSE. **(A)** Mean absolute SHAP values (feature importance) for the RULM model. **(B)** Mean absolute SHAP values for the HFMSE model. **(C,D)** SHAP summary plots showing the distribution of SHAP values for each feature; colors denote feature values from low (blue) to high (pink). Ulnar CMAP amplitude ranked highest for RULM, whereas CK ranked highest for HFMSE. Higher ulnar CMAP values were generally associated with higher predicted RULM, and higher CK often increased predicted HFMSE, although contributions of CK varied widely across observations, reflecting between-patient heterogeneity. Models represent the best-performing random forests selected under leave-one-subject-out (LOSO) cross-validation with fold-internal preprocessing. SHAP values reflect contributions to model predictions and do not imply causality. SHAP, SHapley additive explanations; RULM, revised upper limb module; HFMSE, Hammersmith functional motor scale expanded; CK, creatine kinase; Cr, serum creatinine; %VC, vital capacity; CMAP, compound muscle action potential.

The predictive capacity of biomarkers was further evaluated using Ridge, Elastic Net, and Random Forest models under LOSO cross-validation with fold-internal preprocessing. Random Forest showed the best performance (RULM: *R*^2^ = 0.743, RMSE = 6.43 points; HFMSE: *R*^2^ = 0.691, RMSE = 11.11 points) (Supplementary Figure S1). SHAP analysis indicated that ulnar CMAP amplitude was the most important predictor for RULM, followed by CK and %VC. For HFMSE, CK was the strongest contributor, followed by Cr and %VC. SHAP distributions suggested a generally positive relationship between higher ulnar CMAP amplitude and predicted RULM. For HFMSE, CK showed a positive but heterogeneous effect across individuals (Figure 5).

#### 3.3.4 Predictive analysis of treatment response

Baseline variables were used to model 9-month change in motor scores. Nested cross-validation demonstrated poor predictive performance of Random Forest models (ΔRULM: *R*^2^ = −6.14, ΔHFMSE: *R*^2^ = −1.12). Negative R^2^ values indicate that model performance below that of the mean-based baseline predictor.

## 4 Discussion

This longitudinal study of 23 Japanese adult patients with genetically confirmed SMA characterized clinical, genetic, and biomarker profiles over extended follow-up. The findings highlight the heterogeneity of clinical severity associated with *SMN2* copy number and the presence of hybrid alleles, and suggest that accessible biomarkers such as %VC, CK, Cr, and ulnar CMAP amplitude may serve as indicators of motor function. However, despite these associations, the evaluated biomarkers did not demonstrate predictive utility for short-term treatment responsiveness to nusinersen.

A notable finding in this cohort was the diversity of *SMN2*-related genotypes. Hybrid *SMN* alleles, in which exon 7 originates from *SMN2* and exon 8 from *SMN1*, were detected in 8 of 23 patients (35%), including one patient with two hybrid copies. In Japan, the reported prevalence of hybrid *SMN* genes is relatively high, with one study documenting a frequency of 10.8% (24 of 204 individuals), although regional variation has been described (9). Prior studies have suggested that genotypes containing hybrid *SMN* genes are more frequently associated with SMA types II and III than those without such genes. The relatively high proportion of hybrid carriers in our cohort likely reflects the inclusion of only patients with SMA types II or III (9, 22).

In our cohort, a greater total number of *SMN2*-related copies, including hybrid alleles, was associated with later symptom onset, higher motor function scores, and more favorable biomarker profiles, consistent with a milder phenotype. Physiological, electrophysiological, and laboratory measures-particularly %VC and ulnar CMAP amplitude-showed consistent trends across genotype strata and may represent practical indicators of motor status. However, subgroup sizes, particularly those defined by hybrid alleles, were small, limiting precision and warranting caution in interpretation. Overall, these findings are consistent with prior reports linking higher *SMN2* copy number to attenuated disease severity (1, 2).

When stratified by the presence of hybrid gene status, patients with four *SMN2* copies who carried at least one hybrid allele exhibited higher RULM and HFMSE scores, suggesting a milder phenotype. In contrast, among patients with three copies, the presence of hybrid alleles appeared to be associated with greater severity. However, these findings should be interpreted with caution, as potential confounding factors may have influenced the results. In particular, sex differences may partly explain the observed pattern, given prior evidence that female patients tend to have milder phenotypes (23). In our cohort, sex distribution differed between subgroups, which may have contributed to the apparent discrepancy. Previous studies examining genotype–phenotype relationships have reported inconsistent findings; some suggest that hybrid *SMN* genes confer a milder phenotype than canonical *SMN2* genes, whereas others found no significant difference (9, 24, 25). Larger studies are required to clarify these associations.

Long-term follow-up indicated that although some patients experienced modest motor improvements after initiation of nusinersen, these gains tended to plateau after approximately 15 months. Among the 18 patients with both baseline and 9-month follow-up data, one-third showed improvement, one-third remained stable, and one-third showed decline. In a progressive disease such as SMA, stability itself may represent a meaningful therapeutic effect (26). However, the attenuation of treatment effects over time raises important questions regarding treatment duration, dosing intervals, and optimization strategies in adult patients. Notably, the Japanese administration schedule (loading doses at months 0, 1, and 3 followed by maintenance every 6 months) differs from the more frequent maintenance intervals used in the United States and Europe (10, 11). Whether more intensive dosing could improve long-term outcome in adult patients remains to be determined (27).

The identification of accessible biomarkers correlated with motor function represents an important aspect of this study. Both conventional statistical modeling and machine-learning approaches identified %VC, CK, Cr, and ulnar CMAP amplitude as key contributors to motor function scores. SHAP analysis further suggested that ulnar CMAP amplitude had the strongest influence on RULM, whereas CK contributed more prominently to HFMSE. These findings may reflect difference in the domains captured by each motor scales: RULM primarily assesses distal upper limb function and may be more influenced by peripheral nerve and motor unit integrity, whereas HFMSE reflects broader motor capacity, including proximal and axial function, and may be more sensitive to systemic factors. Importantly, %VC, CK, Cr, and ulnar CMAP amplitude are all readily available in routine clinical practice, supporting their potential utility as pragmatic markers of disease status. In contrast, other proposed SMA biomarkers, such as neurofilament light chain in serum or CSF, interleukin-8, and cathepsin D, although promising, remain limited by technical and logistical constraints (28–35).

Several limitations of this study should be acknowledged. First, the sample size was small, and subgroup analyses within each genotype category lacked sufficient statistical power. Second, all participants were adults, limiting generalizability of the findings to pediatric populations or to patients with SMA type I. Third, although multiple biomarkers were evaluated, additional modalities such as advanced electrophysiology or quantitative MRI may provide further insights (36). Finally, as a single-center study, external validation in larger multicenter cohorts is required.

## 5 Conclusion

This longitudinal study of Japanese adults with SMA demonstrated significant genotype–phenotype correlations and identified clinically accessible biomarkers that associated with motor function. Although these markers may aid in disease monitoring, their ability to predict treatment responsiveness appears limited in adult patients. The findings underscore the need for further research to refine prognostic markers and optimize therapeutic strategies in adult SMA.

## Data Availability

The raw data supporting the conclusions of this article will be made available by the authors, without undue reservation.
