# Supplementary material for: Longitudinal motor function and biomarker correlates in treated adult spinal muscular atrophy: a single-center cohort study
Source: Front Neurol. 2026 Jun 11;17:1870339. doi: 10.3389/fneur.2026.1870339 (PMC13295155; doi:10.3389/fneur.2026.1870339)
Supplement: Supplementary file 1 [file Supplementary_file_1.docx]

Supplementary information:

Genotype–Phenotype Correlations and Candidate Biomarkers in Adult Spinal Muscular Atrophy: A Longitudinal Cohort Study

**Patient 6.**

The patient noticed gait abnormalities in early childhood. By approximately age 6 he consistently finished last in footraces, and by age 12 he experienced frequent falls. At age 13 he was diagnosed with spinal muscular atrophy (SMA) at a university hospital. Motor function gradually declined; he could no longer climb stairs by age 18 and transitioned to wheelchair use at age 20. Genetic testing showed a homozygous SMN1 deletion and three SMN2 copies.

Nusinersen was initiated at age 29. Baseline scores were RULM 37 and HFMSE 33. Although he reported feeling “lighter” after the first dose, scores were unchanged at the time of the second injection (RULM 37, HFMSE 33). Minimal changes were observed at the third (RULM 36, HFMSE 35) and fourth (RULM 35, HFMSE 33) doses. Despite continued dosing every six months, a gradual decline occurred, reaching RULM 35 and HFMSE 30 by the eighth injection (≈2 years after initiation).

Because hospital admissions were restricted during the COVID-19 pandemic, he switched to risdiplam after the ninth nusinersen injection. During oral therapy he experienced persistent mild diarrhea and ultimately elected to return to nusinersen due to intolerance; at that time, scores had declined to RULM 29 and HFMSE 25. After re-initiating nusinersen, scores improved to RULM 33 and HFMSE 29 at 4 weeks, and to RULM 36 and HFMSE 30 at 6 months, suggesting partial recovery.


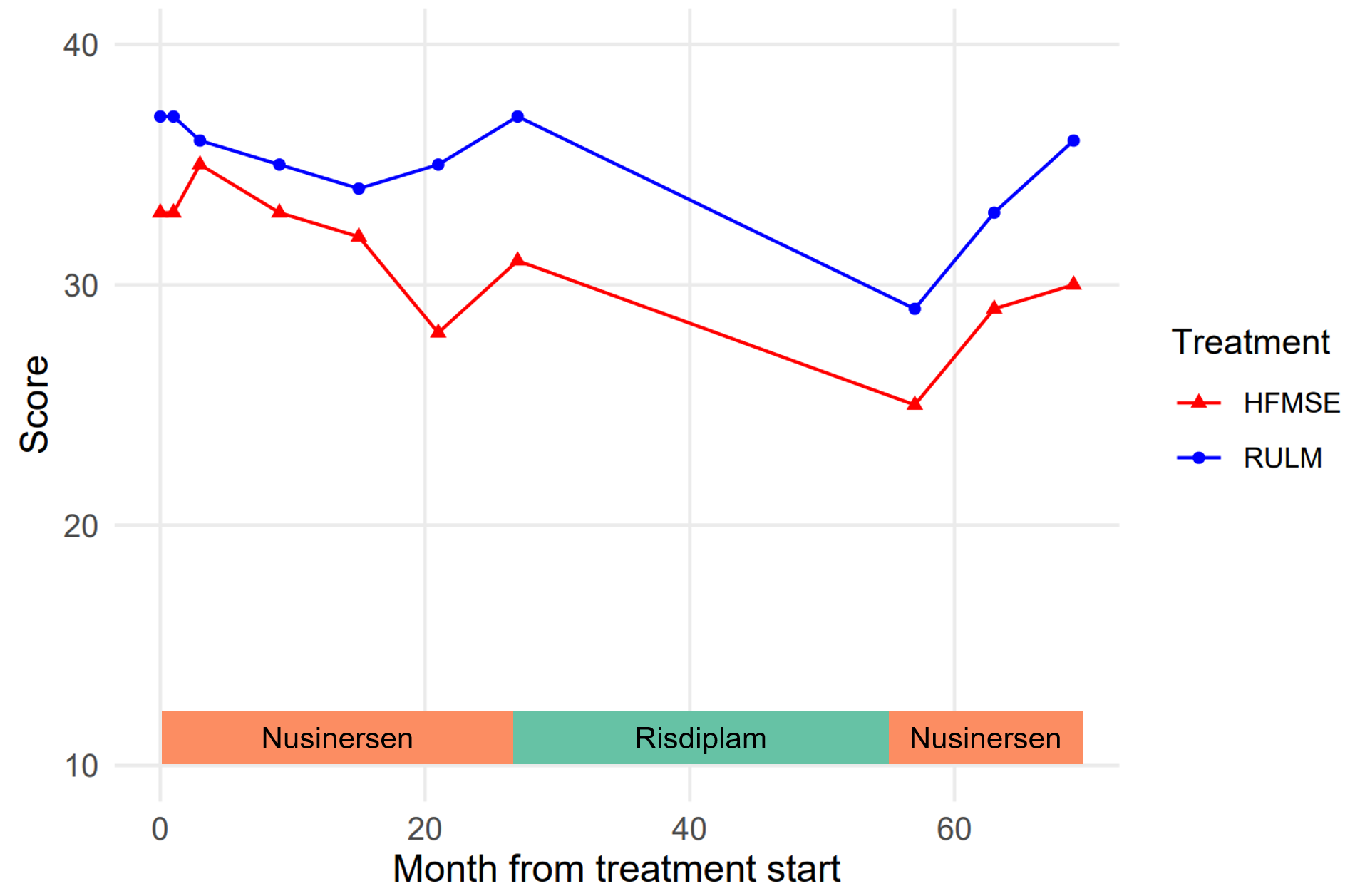


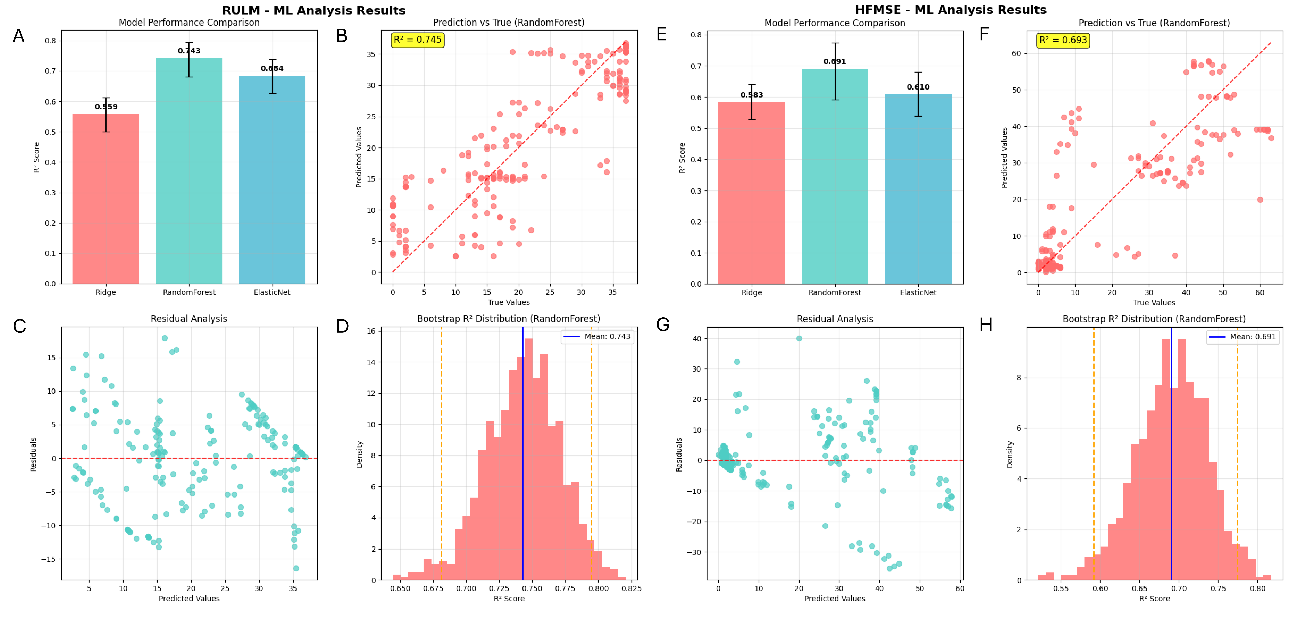


**Supplementary Figure S1. Machine-learning model performance and diagnostics for predicting motor function.**
Panels A–D depict results for RULM; panels E–H depict results for HFMSE. (A, E) Mean out-of-fold coefficient of determination (R²) with 95% confidence intervals under leave-one-subject-out cross-validation (LOSO); among the evaluated algorithms, Random Forest achieved the highest performance. RULM: R²=0.743 (95% CI, 0.681–0.974); RMSE=6.43 (95% CI, 5.79–7.06) points; HFMSE: R²=0.691 (95% CI, 0.591–0.774); RMSE= 11.11 (95% CI, 9.56–12.70) points. (B, F) Predicted versus observed scores for the Random Forest models, with R² computed on held-out subjects; each point represents one observation. (C, G) Residual distributions for the Random Forest models on held-out subjects. (D, H) Bootstrap distributions (1,000 resamples) of out-of-fold R² for the Random Forest models; the vertical line denotes the bootstrap mean and the dashed lines the 95% percentile interval.

Abbreviations: RULM, Revised Upper Limb Module; HFMSE, Hammersmith Functional Motor Scale Expanded. RMSE, Root Mean Squared Error.

**Supplementary Methods**

Software and overall approach

Analyses were performed in R 4.3.2 (RStudio) and Python 3.11 (Google Colaboratory). Correlations were computed in R; linear mixed-effects models (LMM) and machine-learning (ML) models were implemented in Python. Because the dataset contains repeated measurements per patient, we used methods that account for within-patient correlation and emphasized effect sizes with 95% confidence intervals rather than relying only on *p*-values.

**1) Correlation analysis**

We first described simple relationships between motor scores—Revised Upper Limb Module (RULM) and Hammersmith Functional Motor Scale Expanded (HFMSE)—and candidate laboratory/physiological/electrophysiological measures:

- %VC (slow vital capacity, reported as percent predicted; seated spirometry with nose clip)
- Serum biomarkers: creatine kinase (CK), serum creatinine (Cr), and creatine
- Ulnar nerve compound muscle action potential (CMAP) amplitude and ulnar decremental response on 3 Hz repetitive nerve stimulation
- 6-minute walk test (6MWT) for ambulant patients

We used Pearson correlation coefficients with pairwise deletion (pairwise-complete observations) so that missingness in one variable did not drop entire rows from all pairwise analyses.
*Note:* 6MWT is itself a motor-performance scale; it was summarized descriptively and in correlations but not used later as a predictor of motor scores.

**2) Linear mixed-effects models (LMM)**

Purpose

To identify independent predictors of motor function while accounting for repeated measures within the same person, we fit separate LMMs for RULM and HFMSE.

Model structure

- A random intercept per patient separated between-person differences from within-person trajectories.
- Fixed-effect predictors were prespecified from the correlation matrix: variables with an absolute correlation ≳ 0.4 with either RULM or HFMSE were retained—%VC, CK, Cr, creatine, and ulnar CMAP amplitude.
- We deliberately did not use RULM to predict HFMSE (or vice versa).
- Month since baseline was mean-centered; treatment (nusinersen/risdiplam) was included as an adjustment covariate only (no interactions were modeled).

Data handling for LMM

Within each patient and along the time axis (Month), predictor gaps were addressed in this order: 1) linear interpolation → 2) forward fill → 3) backward fill.
Values still missing after this sequence remained missing and were excluded listwise for that specific model. Outcomes (RULM, HFMSE) were not imputed in primary analyses.

Estimation and reporting

Models were fit with statsmodels using restricted maximum likelihood (REML); if the default optimizer struggled, a fallback (e.g., Nelder–Mead) was used. We report fixed effects as β (SE), Wald *z*, two-sided *p*, and 95% CI. For completeness, the between-patient variance (“Group Var”) and residual variance (“Scale”) were recorded but not emphasized in main tables.

**3) Machine-learning models**

Evaluation frameworks (overview)

We used two complementary cross-validation frameworks:

- Concurrent score prediction (RULM/HFMSE at observed visits): leave-one-subject-out (LOSO) CV at the patient level; all preprocessing and any tuning were performed within each training fold to prevent leakage; performance reported as out-of-fold for R^2^, RMSE (points), and MAE (points).
- Short-term change prediction (Δ at 9 months): nested CV with an outer five-fold loop and inner grid search; performance summarized as mean ± SD across outer folds for R^2^, RMSE (points), and MAE (points).

Goals and features (concurrent score prediction)

We trained supervised regressors (Ridge, Elastic Net, Random Forest) to predict RULM and HFMSE and to explore variable contributions. We started from the within-patient–imputed dataset described above. Candidate features mirrored the clinical/statistical set and, where available, included ulnar decrement. 6MWT was excluded as a predictor. Patients contributed if they had ≥ 3 outcome time points; rows with missing outcomes were excluded.

Preventing information leakage (both frameworks)

To avoid optimistic estimates, all preprocessing was confined to training data in each split:

- Median imputation of missing predictors → standardization → model fitting, wrapped in a Pipeline inside the training partition.
- For LOSO, any hyperparameter tuning was conducted with grouped inner CV on the training data only.
- For nested CV, hyper-parameter selection was restricted to the inner loop; the held-out outer fold was untouched until final evaluation.

Models and metrics

We evaluated Ridge, Elastic Net, and Random Forest regressions. For LOSO, performance on held-out subjects was summarized by R^2^, RMSE (points), and MAE (points), with 95% CIs obtained from 1,000 bootstrap resamples of out-of-fold predictions.

Model interpretation

For Random Forests, we used SHAP (SHapley Additive exPlanations) to quantify each feature’s contribution (mean absolute SHAP values) and visualize how low vs high values influenced predictions (summary plots). SHAP was preferred over impurity-based importances to mitigate biases under correlated features. *(SHAP reflects contribution to model prediction and does not imply causality.)*

Change-over-time (9-month) prediction

We applied a dedicated ML framework to predict short-term change from baseline:

- Outcome construction: the 9-month change was operationalized as the first post-loading motor assessment after nusinersen initiation (9 months). Each patient contributed one row; outcomes were not imputed.
- Predictors (baseline only): %VC, CK, Cr, creatine, ulnar CMAP amplitude, ulnar decrement, plus the corresponding baseline motor score (RULM for ΔRULM; HFMSE for ΔHFMSE).
- Evaluation: nested cross-validation (outer five-fold; inner grid search over Random Forest hyperparameters such as *n_estimators* and *max_depth*), with median imputation and scaling fit within training folds. Performance was summarized as mean ± SD across outer folds for R^2^, RMSE (points), and MAE (points). *(Note:* R^2^ *can be negative when a model underperforms the mean-predictor baseline.)*

Units and naming

- %VC: percent predicted (%); CK: U/L; Cr: mg/dL; creatine: mg/dL; ulnar CMAP amplitude: mV; 6MWT: meters.
